# Acute esophageal necrosis after kidney transplantation: A case report

**DOI:** 10.1097/MD.0000000000024623

**Published:** 2021-02-12

**Authors:** Na Young Kim, Yoo Jin Lee, Kwang Bum Cho, Kyubok Jin, Ju Yup Lee

**Affiliations:** a Division of Gastroenterology; b Nephrology, Department of Internal Medicine, Keimyung University School of Medicine, Daegu, Korea.

**Keywords:** acute esophageal necrosis, black esophagus, hematemesis, kidney transplantation

## Abstract

**Rationale:**

Acute esophageal necrosis (AEN) is a rare syndrome with characteristic endoscopic and pathologic findings. It usually results from a combination of tissue hypoperfusion, impaired local defense barriers, and massive reflux of gastric contents. We report a case of AEN after a kidney transplant.

**Patient concerns:**

A 53-year-old man with hypertension and end-stage renal disease presented with abdominal pain and a single episode of hematemesis 14 days after kidney transplantation.

**Diagnosis:**

Upper endoscopy revealed circumferential black coloration in the mid to lower esophageal mucosa. Esophageal biopsy showed ulcer, and immunostains were negative for viral etiology.

**Interventions:**

Conservative management was done with total parenteral nutrition and proton pump inhibitor.

**Outcomes:**

The patient experienced no further episodes of hematemesis or abdominal pain and follow-up endoscopy showed remarkable changes from the black mucosa to a red friable mucosa with whitish exudates.

**Lessons:**

In the case, AEN occurred in the setting of normal blood pressure after major surgery despite the absence of preceding factors such as hypotension and infections. The possibility of AEN should be considered in patients with solid organ transplantation who present with abdominal pain, dysphagia, and hematemesis.

## 1 Introduction

Acute esophageal necrosis (AEN), also known as “black esophagus” or “acute necrotizing esophagitis,” is a very rare disease that can be diagnosed by characteristic endoscopic and pathologic findings. AEN is defined endoscopically as dark pigmentation of the esophagus with histological mucosal necrosis. The exact pathophysiology is unclear; it is likely multifactorial and usually results from a combination of tissue hypoperfusion, impaired local defense barriers, and massive reflux of gastric contents.^[1]^ There have been very few cases of AEN after kidney transplantation. We report a case of AEN in a 53-year-old man after kidney transplant. Informed consent was obtained from the patient for the publication of this case report.

## 2 Case presentation

A 53-year-old man was admitted to our medical center for kidney transplantation. Past medical history was significant for hypertension and end-stage renal disease due to chronic glomerulonephritis; the patient had therefore been on hemodialysis for 11 years. Medication included nifedipine, carvedilol, and entecavir for chronic hepatitis B.

He underwent a kidney transplantation from a brain-dead patient on the day of admission, and simulect (basiliximab) 20 mg induction was administered. Before transplantation, CMV immunoglobulin M (IgM) of the patient and donor was negative, and CMV-immunoglobulin G (IgG) of those was positive. No prophylactic ganciclovir treatment was done. There was no event of intra-operative hypotension including massive bleeding during surgery. Fever or abdominal symptoms were not observed after surgery, and prophylactic antibiotics were discontinued 3 days after surgery. Triple immunosuppressive therapy was administered with oral tacrolimus (FK506) 11 mg, mycophenolate mofetil 1440 mg, and prednisolone 20 mg following kidney transplantation. The serum tacrolimus trough level was maintained between 4 and 10 ng/dL.

**Figure 1:**
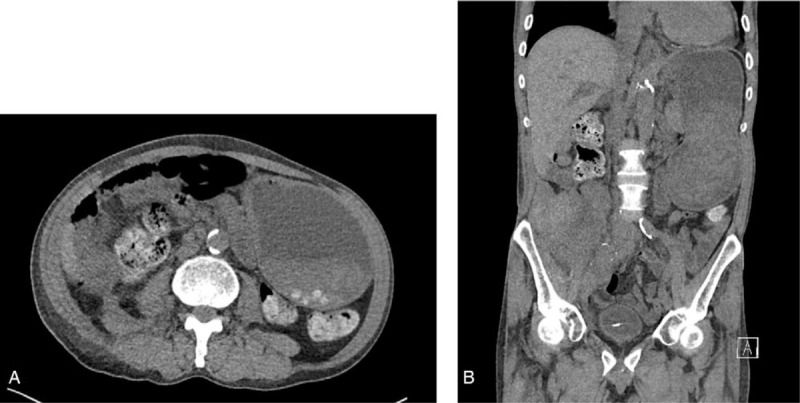
Computed tomography. There was no structural abnormality other than gastric distention.

On day 14 post-transplantation, the patient complained of severe abdominal pain and bloating. Physical examination showed a moderately distended abdomen with mild epigastric tenderness and a temperature of 37.8°C. Laboratory studies revealed the following: white blood cell count, 18,250/μL (neutrophils 84.9); hemoglobin, 9.0 g/dL; platelet count 120,000/nL, C-reactive protein, 0.4 mg/dL (normal <0.5 mg/dL). Computed tomography (CT) was performed, and there was no structural abnormality other than gastric distension (Fig. 1).

**Figure 2:**
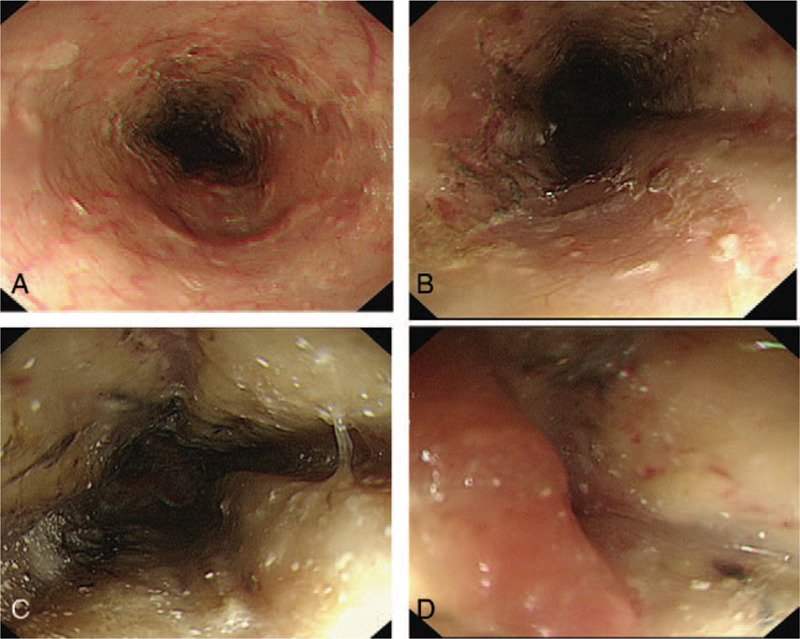
Endoscopic findings 16 days post-transplantation. Endoscopic imaging shows a diffuse black esophageal mucosa that preferentially affects the distal esophagus and stops abruptly at the gastroesophageal junction. (A) Upper esophagus, (B) Mid-esophagus showing area of necrosis. (C) Esophageal mucosa with a circumferential black pigment in the middle-lower esophagus, (D) Gastroesophageal junction.

In the assessment for gastroparesis, a nasogastric tube was inserted for decompression, and the patient was managed with oral intake restriction, intravenous hydration, and parenteral nutrition. After 2 days of management and 16 days post transplantation, the patient developed vomiting and a single episode of hematemesis. Upper endoscopy revealed circumferential black pigmentation, and edematous mucosa covered by exudates was noted at the mid-lower esophagus (Fig. 2). The gastroesophageal junction (GEJ) was intact and the gastric mucosa was normal in direct vision; on retrovision, the duodenum and bulb were unremarkable. The biopsy of the mid-esophagus showed an ulcer, and immunohistochemistry showed negative cytomegalovirus (CMV), herpes simplex virus (HSV-1), and HSV-2 polymerase chain reaction findings.

The patient was treated with a proton pump inhibitor twice a day (intravenous pantoprazole, 40 mg/day) and antibiotics including ceftriaxone and metronidazole were started due to fever. Mycophenolate mofetil was reduced to 1080 mg and oral drugs were changed to injectables, except for immunosuppressants.

**Figure 3:**
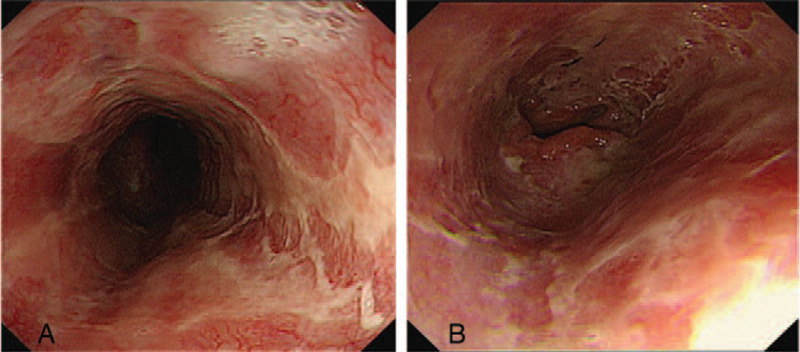
Endoscopic findings 23 days post-transplantation. The black necrotic tissue had changed to a red friable mucosa with erosion. (A) Mid esophagus, (B) Lower esophagus.

**Figure 4:**
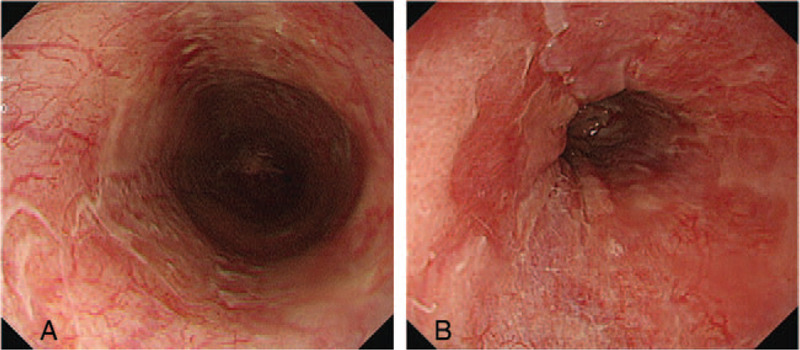
Endoscopic findings 30 days post-transplantation. Erosions had disappeared, and a mild erythematous mucosa was noted in the mid-lower esophagus. (A) Middle esophagus, (B) Lower esophagus.

Follow-up endoscopy 1 week later showed remarkable changes from the black mucosa to a red friable mucosa with whitish exudates, even though circumferential erosions were still seen on the upper esophagus (Fig. 3). The patient experienced no further episodes of hematemesis or abdominal pain. The vital signs and clinical condition stabilized, and the patient was started on a liquid diet. The third endoscopy performed on day 30 post-transplantation showed an overall lesion improvement (Fig. 4). Intravenous antibiotics were discontinued, and the patient resumed a soft diet and was discharged without complications.

## 3 Discussion

AEN was first described in 1990 by Goldenberg et al.,^[2]^ with an incidence of 0.01% to 0.2% in patients undergoing esophagogastroscopy.^[3]^ The prognosis is poor, and severity largely depends on the patient's general state of health. The mortality rate specific to AEN is ∼6%.^[4]^

The clinical presentation is prominent with upper gastrointestinal (GI) bleeding, with most patients presenting with hematemesis or melena. Other symptoms may include abdominal pain, nausea, vomiting, and dysphagia. AEN is characterized by endoscopic imaging of the diffuse black esophageal mucosa that preferentially affects the distal esophagus and stops abruptly at the GEJ. Biopsy is not required to establish a diagnosis. Biopsy is for the differential diagnosis of melanosis,^[5]^ pseudomelanosis,^[6]^ acanthosis nigricans,^[7]^ melanoma,^[8]^ and other caustic and corrosive agents that may induce necrosis. Histopathology usually shows necrotic debris, mucosal, and submucosal necrosis with local inflammatory response.^[1]^

There is no specific therapy for AEN. The current recommendation is systemic resuscitation with intravenous fluid therapy and proton pump inhibitors until there is an improvement in clinical status. Bowel rest and adequate nutrition through total parenteral nutrition are essential to ensure effective healing of the mucosa. In our case, a nasogastric tube was inserted for decompression before the endoscopic test for the assessment of gastroparesis. However, there is a risk of perforation due to impaired mucosal membrane, and nasogastric tube insertion should be avoided. Prophylactic antibiotics are not necessary. Empirical broad-spectrum antibiotics should be initiated in cases of fever, suspected esophageal perforation, or immune compromise. Surgical intervention is reserved for a perforated esophagus resulting in mediastinitis and abscess formation.

Possible complications of AEN include perforation, stricture, and motor abnormalities. Perforation is a life-threatening complication with an incidence of approximately 5%.^[9]^ Esophageal stricture, which results in significant dysphagia, can be seen in ∼10% of patients undergoing follow-up esophagogastroduodenoscopy.^[9]^

The pathophysiology usually involves a combination of esophageal ischemia, reflux injury from gastric contents, and impaired mucosal local defense barriers.^[1,4,10,11]^ It may arise in cases of hemodynamic compromise, diabetic ketoacidosis, alcoholic ketoacidosis, esophageal local infection, solid organ transplantation, postoperative status, or acute gastric outlet obstruction. Several drugs have been involved in this pathophysiology of AEN, including non-steroidal anti-inflammatory drugs (NSAID), antihypertensive agents, terlipressin, clozapine, olanzapine, cocaine, and Immunosuppressant agent.^[1,12]^ Esophageal ischemia may be induced by vasoconstrictive agents such as terlipressin, and antihypertensive agents which induce transient low circulatory flow. Immunosuppressant agents like Tacrolimus and MMF compromise gastrointestinal mucosa ability to local defense barrier function and increase the risk of opportunistic infection such as CMV esophagitis.^[12,13,14]^

In reported case of Tacrolimus-induced AEN,^[15]^ there was no response in general conservative treatment, and improvement was observed after discontinuation of tacrolimus. In our case, the patient maintained triple immusuppressant drugs and showed improvement with conservative treatment, and did not take other triggering drugs such as NSAIDs and antipsychotics.

After kidney transplantation, intravenous hydration was performed for net balance of daily Input/Output (I/O), but urine volume increased as kidney function improved, negative I/O 1000 to 2000 mL was checked until 10 days postoperatively. Our patient may have been in a state of dehydration after the surgery, and the wound caused a decrease in esophageal pressure. Peripheral circulatory failure also decreases gastric motility and distention. Because of decreased gastric motility, large volumes of gastric contents continuously covered the esophagus for a prolonged period, impairing the normal defense mechanisms of the esophagus and resulting in mucosal necrosis.

There have been some cases of AEN after kidney transplantation, including primary CMV infection^[16]^ and intraoperative cardiac arrest with associated hypotension.^[17]^ But in the case, there was no event of intra-operative hypotension including massive bleeding during surgery, and biopsy and immunohistochemistry for CMV was negative. Our patient developed AEN without an episode of hypotension or infectious etiology. Despite the absence of preceding factors such as hypotension and infections, AEN may occur in the setting of normal blood pressure after major surgery. This should raise the clinicians’ awareness of the possibility of AEN in patients post-transplant presenting with abdominal pain, bloating, and hematemesis.

## 4 Conclusions

AEN is a very rare syndrome that can cause fatal complications. The possibility of AEN should be considered in patients with solid organ transplantation who present with abdominal pain, dysphagia, and hematemesis. Diagnosis is established by the presence of a striking endoscopic image of a black distal esophagus with abrupt transition at the GEJ. After diagnosis, symptomatic treatment should be performed with a proton pump inhibitor, and attention should be paid to the occurrence of serious complications such as esophageal perforation, mediastinitis, and abscess formation.

## Author contributions

**Conceptualization:** Kyubok Jin, Ju Yup Lee.

**Supervision:** Yoo Jin Lee, Kwang Bum Cho, Ju Yup Lee.

**Writing – original draft:** Na Young Kim.

## Abbreviations

Abbreviations: AEN = acute esophageal necrosis, CMV = cytomegalovirus, CT = computed tomography, GEJ = gastroesophageal junction, HSV = herpes simplex virus.
